# Food insecurity and its impact on glycaemic control in diabetic patients attending Jabulani Dumani community health centre, Gauteng province, South Africa

**DOI:** 10.4102/phcfm.v13i1.2906

**Published:** 2021-05-25

**Authors:** Kayumba B.A. Nsimbo, Neetha Erumeda, Deidre Pretorius

**Affiliations:** 1 Division of Family Medicine, School of Clinical Medicine, University of the Witwatersrand, Johannesburg, South Africa

**Keywords:** food insecurity, glycaemic control, Type 2 diabetes, prevalence, adherence

## Abstract

**Background:**

To the best of our knowledge no studies have been conducted to assess the relationship between food insecurity and poor glycaemic control in diabetic patients in peri-urban settings of the South African context.

**Aim:**

The study aimed to assess food insecurity and its relationships with glycaemic control and other patient characteristics amongst diabetic patients attending Jabulani Dumani Community Health Centre.

**Setting:**

The study was conducted in a primary healthcare facility in the south sub-district of Ekurhuleni health district, the Gauteng province, South Africa.

**Methods:**

This was a cross-sectional descriptive study involving 250 patients. Data were collected by using an interview-administered Household Food Insecurity Access Scale questionnaire. Descriptive and inferential statistical analyses by using Stata 14.0 statistical software were performed. Chi square and logistic regression tests assessed the association between socio-demographic characteristics, glycaemic control and food insecurity.

**Results:**

Amongst 250 recruited participants, 82.4% were above 50 years, 64% women, 88.8% South African citizens and 42.4% had a household size of ≥ 5 people. Sixty-four percent and 69.9% were classified as having food insecurity and poor glycaemic control, respectively. On further analysis, food insecurity was associated with unemployment (adjusted odds ratio [AOR] = 2.94; 95% confidence interval [CI]: 1.51–5.75), being a South African citizen (AOR = 1.60; 95% CI: 0.66–3.86), household size of ≥ 5 people (AOR = 1.77; 95% CI: 0.98–3.19) and uncontrolled glycaemic level (AOR = 5.38; 95% CI: 2.91–9.96).

**Conclusion:**

Food insecurity in diabetic patients constitutes a serious challenge for glycaemic control. It is critical for healthcare providers in primary care settings to ensure screening for early identification and management of food insecurity and take measures to prevent poor glycaemic control.

## Introduction and background

Diabetes mellitus constitutes a serious public health problem worldwide, including developing countries in Africa.^1^ It also accounts for significant morbidity and mortality worldwide and is the second leading cause of death in South Africa.^2,3,4^ Based on the most recent report of the International Diabetes Federation (IDF), in 2015 there were 14.7 million diabetics in African countries, and South Africa accounted for 3.8 million cases, which represented 7% of its overall population.^5^ In 2010, the prevalence of diabetes in the Republic of South Africa was estimated at 4.5% compared with 7% in 2015.^6^ These data show that the prevalence of diabetes has nearly doubled. This increase in its prevalence is mainly because of the lifestyle changes of the population, including urbanisation, unhealthy diet and a decrease in physical activity.^7,8^

The primary purpose in the management of diabetes is to reduce blood glucose to prevent complications such as acute coronary syndrome, peripheral artery disease, chronic kidney disease and diabetic ketoacidosis.^9^ Many patient-related factors predispose diabetic patients to poor glycaemic control such as poor knowledge of diabetes, low level of education and poor compliance with treatment.^10^ Amongst these patient-related factors mentioned here, food insecurity has been postulated to be one of the potential patient-related factors that predispose diabetic patients to poor glycaemic control.^11^

Households are food-insecure whenever the availability of adequate nutrition and safe foods or the ability to obtain food within the social legal norms is limited or doubtful.^12.^ Food insecurity affects low-, middle- and high-income countries.^13^ In South Africa, the unemployment rate keeps increasing (28.1%),^14^ and the society is also characterised by socio-economic disparities, which may contribute to food insecurity within its households.^14^ It has been estimated in 2019 that 20% of South African households were food-insecure.^15^

In the primary healthcare setting of South Africa, clinicians may not be aware of whether their patients can afford or can access appropriate food on a regular basis. Their patients might experience food insecurity, which may potentially affect the overall health outcome of the disease. Previous studies have been conducted in developed and developing countries, but no studies have been conducted in the primary healthcare facilities in a peri-urban South African setting. Hence, this study aimed to assess the proportion of food insecurity and its relationships with glycaemic control amongst diabetic patients attending Jabulani Dumani Community Health Centre (CHC).

## Research design and methods

### Design and setting

This was a cross-sectional study conducted at Jabulani Dumani CHC, Vosloorus, in the municipality of Ekurhuleni, the Gauteng province, in South Africa, with a catchment population of 60 436 at the time of the study. The facility provides a range of curative services, both acute and chronic, as well as preventive, health promotion and rehabilitation services. According to statistics of the City of Ekurhuleni, 4% of its population has no education, 41% of the population in the city have matric qualification. The unemployment rate in Ekurhuleni is 31.8%, and over 35.9% of the population lives in poverty.^16^

### Population and sampling strategy

The sample of this study was extracted from 2950 diabetic patients attending Jabulani Dumani CHC.

All adult diabetic patients who were 18 years and older, on diabetic treatment for at least one year, with or without comorbidities or complications and able to give consent were eligible to participate. The diabetic patients who were pregnant or very ill were excluded. Sample size estimation was carried out on nQuery Advisor, Release 7.0 and its calculation was based on a reliable estimation of the glycaemic control rate (percentage) by using the following assumptions.

A proportion of 0.192 (19.2%) of patients have glycaemic control. Accuracy of ±0.05 (5%) was used for an estimation of the glycaemic control rate. With a sample size of 239 patients, a two-sided 95% confidence interval (CI) for the glycaemic control rate would be within ±0.05 (5%) of the control rate that would be calculated from the sample. To allow for a 5% drop rate, a rounded sample size of 250 patients was proposed for this study. A systematic sampling procedure was utilised to select 250 patients. Every third patient was invited to participate in the study. Study participants were recruited between 08:00 and 16:00 on weekdays until the required sample was obtained.

### Measurement tool and data collection

Data were collected by using a self-administered questionnaire, which had two parts: the first part collected information on socio-demographic characteristics, including age, sex, marital status, immigration status, household size, employment status, socio-economic position (SEP) and body mass index (BMI). The second part included a Household Food Insecurity Access Scale (HFIS).^17^ Its validity and adaptability have been tested in developing countries such as Ethiopia and Tanzania, and the scale had good internal consistency.^18,19^ In this study, participants were considered food-insecure if the score was between 10 and 27 and food-secure if the total score was between 1 and 9 according to the tool. The most recent HbA1c available in the participants’ medical records performed in the last 3 months was taken as a measure of glycaemic control. Poor glycaemic control was defined as an HbA1c > 7 for patients below the age of 65 and > 8 for above 65 years.

Two trained research assistants co-facilitated the research process; the assistant who spoke the local language assisted participants to understand the information sheet, consent form and questionnaire. Every third diabetic patient who had completed his or her scheduled follow-up visits with the sister or the doctor was approached by the researcher. A patient information sheet was provided, and the study was explained. If they volunteered to participate and met the inclusion criteria, then a consent form was signed. The researcher accompanied them to a separate room where these questionnaires were completed by the participants to ensure confidentiality. Participants’ information such as HbA1c and BMI were collected from their clinical records and captured in the data collection sheet, which was attached to each of the questionnaires by the researcher.

### Data analysis

Data analysis was carried out by using Stata 14.0 statistical software. Categorical variables were reported as frequencies and percentages. Continuous variables were reported in terms of mean and standard deviation. Association between socio-demographic characteristics, glycaemic control and food insecurity was tested by using the Pearson chi-square test, and logistic regression was used to assess the strength of the associations. The level of statistical significance was set at a CI of 95% and *p*-value of < 0.05.

### Ethical considerations

Ethical approval was obtained from the Human Research Ethics Committee (HREC) of the University of the Witwatersrand (clearance certificate number: M160202). Permission was also obtained from Ekurhuleni Health District Research Committee. Informed consent was obtained from each participant. The questionnaires were completed anonymously and raw data were kept password protected.

## Results

A total of 250 patients participated in the study. Participants’ socio-demographic and biographic characteristics are presented in Table 1. The mean age was 58.67 years (range: 29–88 years). In this study, amongst the participants, 64% were female, 88.8% were South African citizens, 47.6% were married or cohabiting, 76.8% were unemployed and 57.6% had less than five members in their households. The study results also found that 63% of participants were food-insecure, and 69.9% were found to have poor glycaemic control.

**TABLE 1:** Participants’ socio-demographic and biographic characteristics.

Demographic parameters	Frequency	
	*N*	%
**Age group (years) (*n* = 250)**		
< 30	1	0.40
30–39	12	4.80
40–49	31	12.40
50–59	83	33.20
60 or above	123	49.20
**Sex (*n* = 250)**		
Female	160	64.00
Male	90	36.00
**Immigration status (*n* = 250)**		
Citizen	222	88.80
Non-citizen	28	11.20
**Marital status (*n* = 250)**		
Single	43	17.20
Married/cohabiting	119	47.60
Divorced/separated	36	14.40
Widowed	52	20.80
**Employment status (*n* = 250)**		
Unemployed	192	76.80
Employed	58	23.20
**Household size (*n* = 250)**		
Less than 5	144	57.60
Equal or more than 5	106	42.40
**SEP**		
1	14	5.60
2	77	30.80
3	159	63.60
**BMI (kg/m^2^)**		
16–19	5	2.00
20–24	27	10.80
25–29	89	35.60
30–34	76	30.40
35–39	36	14.40
40 or greater	17	6.80
**Food security (*n* = 250)**		
Secure	91	36.40
Insecure	159	63.60
**Glycaemic control (*n* = 250)**		
Controlled	76	30.40
Uncontrolled	174	69.60

SEP1: Low socio-economic position based on asset register.^20,21^

SEP2: Moderate socio-economic position based on asset register.

SEP3: High socio-economic position based on asset register.

SEP, socio-economic position; BMI, body mass index.

Association between socio-demographic and biographic characteristics and food insecurity was tested by using a Chi-square test as shown in Table 2. The results found statistically significant associations between immigration status (*p* = 0.049), household size (*p* = 0.045), employment status (*p* = 0.033) and food insecurity. The study also showed that there was a statistically significant association between glycaemic control and food insecurity (*p* = 0.000). No significant associations were found between age, sex, marital status, SEP, BMI and food insecurity.

**TABLE 2:** Association between socio-demographic and biographic characteristics and food security.

Categories	Odds ratio	*P*-value
**Age group**		
<30 years	1.00	-
30–39 years	0.33	0.524
40–49 years	0.46	0.639
50–59 years	0.76	0.871
>60 years	0.54	0.705
**Immigration status**		
Non-citizen	1.00	-
Citizen	2.22	0.049
**Sex**		
Female	1.00	-
Male	0.85	0.540
**Marital status**		
Single	1.00	-
Married/co-habiting	1.59	0.196
Divorced/separated	1.74	0.236
Widowed	1.79	0.171
**Household size**		
Less than 5	1.00	-
5 or more	1.73	0.045
**Employment status**		
Employed	1.00	-
Unemployed	1.91	0.033
**SEP**		
1	1.00	-
2	0.29	0.123
3	0.27	0.092
**BMI (kg/m^2^)**		
16–19	1.00	-
20–24	1.13	0.900
25–29	1.03	0.976
30–34	1.14	0.887
35–39	1.33	0.769
40 or greater	2.17	0.473
**Glycaemic control**		
Controlled	1.00	-
Uncontrolled	4.79	< 0.001

SEP, socio-economic position; BMI, body mass index.

**TABLE 3:** Predictors of food security.

Risk factor	*N*	%	Unadjusted analysis (*n* = 250)			Adjusted analysis (*n* = 250)		
			OR	95% CI	*p*-value	OR	95% CI	*p*-value
**Employment status**								
Employed	58	23.2	1.00	-	0.033	1.00	-	0.002
Unemployed	192	76.8	1.91	1.05–3.47	-	2.94	1.51–5.75	-
**Immigration**								
Non-South African Citizen	28	11.2	1.00	-	0.049	1.00	-	0.299
South African citizen	222	88.2	2.22	1.00–4.90	-	1.60	0.66–3.86	-
**Household size**								
Less than five members	144	57.6	1.00	-	0.045	1.00	-	0.056
Five or more members	106	42.4	1.73	1.01–2.95	-	1.77	0.98–3.18	-
**Glycemic control**								
Controlled	76	30.4	1.00	-	< 0.001	1.00	-	< 0.001
Uncontrolled	174	69.6	4.79	2.69–8.51	-	5.38	2.91–9.96	-

OR, odds ratio; CI, confidence interval.

Where statistically significant associations were detected, logistic regression was carried out to further assess the strength of the associations. Therefore, factors such as immigration status, household size, employment status and glycaemic control were analysed. The odds of being food-insecure was 2.94 for participants who were unemployed compared with those who were employed (odds ratio [OR]: 2.94; 95% CI: 1.51–5.75; *p* = 0.002).

Participants with poor glycaemic control were 5.38 times more likely to have experienced food insecurity when compared with participants who had good glycaemic control (OR: 5.38; 95% CI: 2.96–9.96; *p* ≤ 0.001).

The odds of being food-insecure were 2.9 for participants who were unemployed compared with those who were employed. Participants with poor glycaemic control were 5.4 times more likely to have experienced food insecurity when compared with participants who had good glycaemic control. Immigration status and household size did not demonstrate any statistically significant association with food insecurity in the multivariate mode.

## Discussion

To the best of our knowledge, this is the first study conducted in Southern Africa that has assessed food insecurity and its relationship with glycaemic control in the patients living with diabetes attending a health facility. Most studies carried out globally were conducted in a hospital setting of developed countries, whereas this South African study focussed on the primary healthcare setting.^22^

In this study, we focussed on determining the proportion of diabetic patients with food insecurity, socio-demographic and biographic factors associated with food insecurity and associations between food insecurity and glycaemic control. The main finding was that the proportion of diabetic patients with food insecurity attending this primary healthcare facility was high (63.6%), which was consistent with studies conducted elsewhere in developing countries.^23,24^ Contradictory to the findings in developing countries, studies performed in high-income countries such as Canada and the United States of America have reported a low proportion of food insecurity in diabetic patients (9.3% and 15%, respectively).^25,26^ The results in this study could thus be because of the fact that the study was conducted in a peri-urban setting and other socio-economic characteristics of our study sample such as low-income level. Many of these higher-income countries have food assistance programmes in place to help diabetic patients that could alleviate food insecurity.

With regard to socio-demographic factors, this study found that the age and sex of participants were not significant factors associated with food insecurity. Hilary et al.^11^ found the same in the United States of America, but Bawadi et al. in Jordan reported that age was a significant factor associated with food insecurity in diabetic patients.^23^ Olson et al., however, postulated that female-headed households in the United States of America were more likely to have experienced food insecurity amongst their participants.^27^

There was a statistically significant association between household size and food insecurity in diabetic patients attending Jabulani Dumani CHC. Household size was reported as a contributing factor to food insecurity in both developing and developed countries.^22,28^ These results cannot be interpreted if one does not also take employment status into account. In this study, employment was generally low, thus causing a lack of steady income. With this in mind, feeding multiple family members on limited income results in food insecurity.

Anecdotally, the perception is that food insecurity mainly presents in informal settlements with large numbers of legal and illegal immigrants. The present study showed that South African citizens were more food-insecure when compared with immigrant participants. This study’s findings were in contrast with previous studies conducted in California by Kasper et al.,^29^ which found that the prevalence of food insecurity amongst legal immigrants (Vietnamese and Cambodians) was much higher when compared with American citizens. This study’s findings can be interpreted in various ways. Immigrants may not have access to clinics, and those having access may have other means of support, which was not explored in this study. For South Africans, we know that the philosophy of *Ubuntu* suggests that South Africans will support each other. The challenge in this country is, however, that African families in South Africa have more members per household than the immigrants, and the country itself has low economic growth. Thus, there is an overall struggle to ‘make ends meet’, and there is just not enough financial support available for all, especially as the majority of the participants were unemployed.

It then came as no surprise that a statistically significant relationship was found between the employment status and food insecurity of a household, which was also confirmed by other studies.^30^ Participants who were unemployed were almost twice as likely to have experienced food insecurity when compared with those who were employed, which is similar to other studies conducted elsewhere.^25,31^ However, within this group of participants who claimed to be unemployed, about a third were still food-secure. This may be supported by the fact that being unemployed does not necessarily mean that participants did not engage in activities that generated income. In conversations with patients during consultations, some shared that they sell things such as vegetables, fruit, sweets, etc. on the street and rent their backyard to other people, whilst others receive groceries on a monthly basis from their children. This also suggested that any form of income or support can alleviate the level of food insecurity in a household.

Most important was the statistically significant relationship between glycaemic control and food insecurity in diabetic patients in this study. Participants with poor glycaemic control were five times more likely to have experienced food insecurity (OR = 5.38). The study conducted by Hilary et al. had similar findings, they reported that the likelihood of poor glycaemic control was about two times more for patients with food insecurity.^11^ This difference could be because this study was conducted in a developing country where participants experienced higher levels of food insecurity compared with developed countries, based on socio-political, socio-economic and cultural differences.^32^ According to the Society for Endocrinology, Metabolism and Diabetes of South Africa (SEMDSA) guidelines, the major focus is on following a healthy diet, which includes low carbohydrate and low fat intake and certain Mediterranean diets that help to control blood glucose in diabetic patients.^9^ In reality, the majority of our diabetic patients are unemployed or without a regular predictable income. Therefore, it is difficult for patients to follow the given dietary instructions, as maize meal forms a staple diet for the impoverished population in South Africa. Limited financial resources also influenced food accessibility. Firstly, these patients struggled to obtain nutritious foods because of the high cost and consequently ended up eating cheaper, high-carbohydrate foods, which most likely contributed to poor glycaemic control in our participants. Secondly, when the accessibility of food poses a problem, this leads to a shortage of food in general or at certain times during a month. It has been documented in a previous study that a shortage of food is associated with anxiety and stress, which in turn is associated with poor self-care behaviour (such as poor adherence) and decreased physical activity.^33^ All of these facts may have contributed to poor glycaemic control.

Almost a quarter of the participants with poor glycaemic control were actually food-secure, which suggested that besides food insecurity as a contributing factor of poor glycaemic control in diabetic patients, there might be other factors that could have affected the results, such as adherence to medication. In the light of this discussion on the results of the study, the researcher thus concludes that the socio-economic climate and its associated levels of unemployment, as well as the predominance of females with multiple dependents in the study setting, can be considered significant factors in food insecurity and poor glycaemic control.

## Limitations

Firstly, as this was a cross-sectional study, causal inferences cannot be concluded. Secondly, all data collected by using a validated food insecurity questionnaire were self-reported, and the participants could have been unwilling to disclose perceived private information. Thirdly, when interpreting the results, the researcher became aware of a few other factors not elicited in the questionnaire. For instance, there were no questions regarding budgeting or financial planning, the existence of a second income or any other financial support, adherence to treatment, diabetic distress or self-management. The sampling of this study was geographically biased to African, low-income or unemployed participants, which may affect the generalisability of the results. The lack of data on educational level of participants could also be considered as a limitation to this study.

## Conclusion

Overall, food insecurity in diabetic patients constitutes a serious challenge; hence, it becomes crucial to address this to minimise the proportion of patients with poor glycaemic control. To achieve this, action must be taken by healthcare workers, community and policymakers. Regular screening for food insecurity by healthcare workers is needed for early identification, which contributes to better glycaemic control. As unemployment contributes significantly to food insecurity, healthcare workers must recognise the challenges faced by diabetic patients with low income in managing their condition. It is time that we revise the policy to establish food assistance programmes for diabetic patients; alternatively, policymakers can motivate communal gardens, which will give not only employment opportunities but also access to food. A multidisciplinary approach should be guiding our actions to address food insecurity whilst keeping in mind other social determinants of health.
